# Neuroimmune Activation and Microglia Reactivity in Female Rats Following Alcohol Dependence

**DOI:** 10.3390/ijms25031603

**Published:** 2024-01-28

**Authors:** Jennifer K. Melbourne, Jessica I. Wooden, Erika R. Carlson, Chinchusha Anasooya Shaji, Kimberly Nixon

**Affiliations:** Division of Pharmacology and Toxicology, College of Pharmacy, The University of Texas at Austin, Austin, TX 78712, USA; jennifer.k.melbourne@gmail.com (J.K.M.);

**Keywords:** ethanol, microglia, sex differences, neuroimmune responses

## Abstract

The rates of alcohol use disorder among women are growing, yet little is known about how the female brain is affected by alcohol. The neuroimmune system, and specifically microglia, have been implicated in mediating alcohol neurotoxicity, but most preclinical studies have focused on males. Further, few studies have considered changes to the microglial phenotype when examining the effects of ethanol on brain structure and function. Therefore, we quantified microglial reactivity in female rats using a binge model of alcohol dependence, assessed through morphological and phenotypic marker expression, coupled with regional cytokine levels. In a time- and region-dependent manner, alcohol altered the microglial number and morphology, including the soma and process area, and the overall complexity within the corticolimbic regions examined, but no significant increases in the proinflammatory markers MHCII or CD68 were observed. The majority of cytokine and growth factor levels examined were similarly unchanged. However, the expression of the proinflammatory cytokine TNFα was increased, and the anti-inflammatory IL-10, decreased. Thus, female rats showed subtle differences in neuroimmune reactivity compared to past work in males, consistent with reports of enhanced neuroimmune responses in females across the literature. These data suggest that specific neuroimmune reactions in females may impact their susceptibility to alcohol neurotoxicity and other neurodegenerative events with microglial contributions.

## 1. Introduction

In any 12-month period, nearly 14% of the U.S. population meets the criteria for the diagnosis of an alcohol use disorder (AUD) [1]. Historically, this percentage has been driven by rates of AUD in men; however, women’s misuse of alcohol and their rates of AUD have been climbing across the last decade (reviewed in [2]). The shrinking gap between rates of AUD in men versus women is due to the 84% increase in AUD diagnoses in women compared to only a 35% increase in men [3]. These data reflect a number of other reports documenting increases in heavy or harmful alcohol use, especially binge pattern drinking among women [4,5,6,7]. An understanding of the neurobiology of AUD has been greatly hindered by sex and gender bias in biomedical research [8,9]. The majority of preclinical research in the alcohol field has utilized male animals, with a dearth of literature exploring the effects of alcohol in females [10]. Furthermore, of the research that has explored sex- or gender-related differences in humans and preclinical models of alcohol use, data on female vulnerability to alcohol-induced effects are mixed [11,12] (for a review, see [2,10,13,14]).

Consuming alcohol excessively can produce cognitive impairments and neurodegeneration [13,15,16,17,18,19,20], with damage largely concentrated in corticolimbic regions, including the hippocampus [21]. In human studies, the loss of volume in these regions [22,23] is thought to reflect neuron death [15,24,25], though shrinkage of neuron somas or dendritic trees may also contribute to this phenomenon [26]. Animal models directly link alcohol and neurotoxicity, but the majority of this work has been conducted in males [25,27,28,29,30,31]. Some preclinical research indicates an increased sensitivity to the neurotoxic effects of ethanol in female animals, while others see more effects in males [10,30,31,32,33,34,35].

Multiple components of the neuroimmune system are impacted by alcohol, including, especially, microglia and the central nervous system (CNS) parenchymal macrophages [36,37,38,39]. Microglia display reactivity, i.e., changes in presentation, including changes in morphology, cytokine levels, surface marker expression, etc., in post-mortem tissue from humans with an AUD, though only males have been studied [40,41]. Rodent and primate models have allowed for a more thorough description of the extent of microglia reactivity and their role in different aspects of alcohol addiction [39,42,43,44,45,46] (see also [37,47] for a review). The interpretation of these observations of a microglial reaction after alcohol exposure has been historically problematic, as any microglia reaction has been equated to a pro-inflammatory, tissue damaging phenotype, which is hypothesized to underlie the neuronal loss seen in AUD [36]. With an improved understanding of microglial biology, it is now apparent that microglia display a wide range of context-dependent responses to a CNS insult, which can often be beneficial or protective in nature [48]. As the field of neuroimmunology has advanced in its understanding of the role of the neuroimmune system across brain disorders, we must also update how we study and interpret the interaction between alcohol and the neuroimmune system [37].

A deeper inspection of data from preclinical models and post-mortem tissue from persons with AUD suggests that microglia are subtly reactive, with little evidence of a classic, proinflammatory phenotype (reviewed in [37]). For example, several studies support that neuroimmune activation occurs with excessive alcohol drinking or AUD, as cytokine levels increase; however, the cellular source of these cytokines cannot be disambiguated, e.g., [49,50,51]. In human post-mortem brain tissue, increased immunoreactivity of microglia-specific markers has been observed, but the morphology remains ramified, which is not consistent with the characteristics of a classic proinflammatory or cytotoxic microglia [40]. In male rats exposed to a 4-day ethanol binge model of alcohol dependence, we previously noted microglial reactivity, indicated by an increased binding of ^3^[H]-PK-11195 to the translocator protein 18 kD found on the outer mitochondrial membrane of reactive glia. Additionally, there was an increased OX-42 immunoreactivity (which binds CD11b/c, a component of complement receptor 3 on microglia) observed in the hippocampus and entorhinal cortex [42,52]. However, markers specific to a proinflammatory microglial phenotype, OX-6 (an antibody against major histocompatibility complex II, OX-6/MHCII) and ED-1 (an antibody against cluster of differentiation 68, ED-1/CD68), were unaltered when examined using immunohistochemistry. An examination of the levels of additional neuroimmune markers in tissue homogenate, including cytokines and growth factors, after ethanol exposure showed that the levels of the anti-inflammatory cytokine IL-10 and growth factor TGF-β were increased 7 days after the last dose of ethanol in the hippocampus, and in the entorhinal cortex the proinflammatory cytokine IL-6 was decreased 2 days after the last dose of ethanol [42]. Further study in microglia ex vivo, i.e., isolated from male rats after alcohol dependence, showed an increase in both pro- and anti-inflammatory cell surface markers as detected by flow cytometry, which suggested a more reparative phenotype [43]. The vast majority of this preclinical work has been conducted in males, with only a small subset describing that microglia appear to react to some extent in females [10,31,32,34,35].

Here, our primary goal was to examine microglial reactivity and neuroimmune protein expression in female rats in a model of alcohol dependence. While there are sex differences and brain-region-specific differences in microglial number, morphology, and their reactions (e.g., [53,54]; for a review, see [48]), we focused on the same corticolimbic regions as in our prior work in adult males [42]. These regions are primary sites of damage in both male and female rats in this model [30]. We further extend our work in this model by examining changes in microglial morphology. Microglia respond to changes in their environment with shifts in cellular morphology (here, we use the term “reactive morphology” to describe microglial reactivity that is specific to a change in morphology) in addition to changes in surface marker expression [55]. More traditional metrics for quantifying microglial reactivity, such as densitometry, cannot distinguish between changes in the number of microglia, their morphology, and the surface expression of the marker utilized [56]. Therefore, we implemented a semi-automated image analysis workflow to quantify changes in microglial morphology and number after alcohol (ethanol) exposure. Next, function-specific markers were used to probe for a proinflammatory microglial phenotype. Finally, the hippocampus and entorhinal cortex were examined for pro- and anti-inflammatory cytokine and growth factor expression, once again choosing factors similar to those in our past work in males.

## 2. Results

### 2.1. Ethanol Binge Data

Subject data from the binge ethanol model were collected, compared between time points, and reported in Table 1. There were no differences in ethanol dose, peak BECs, or peak withdrawal behavior among the ethanol-treated groups. Further, all values were similar to those reported previously for females, as well as males in this model.

### 2.2. Microglia from Adult Female Rats Display a Reactive Morphology after 4-Day Binge Ethanol Exposure

**Figure 1:**
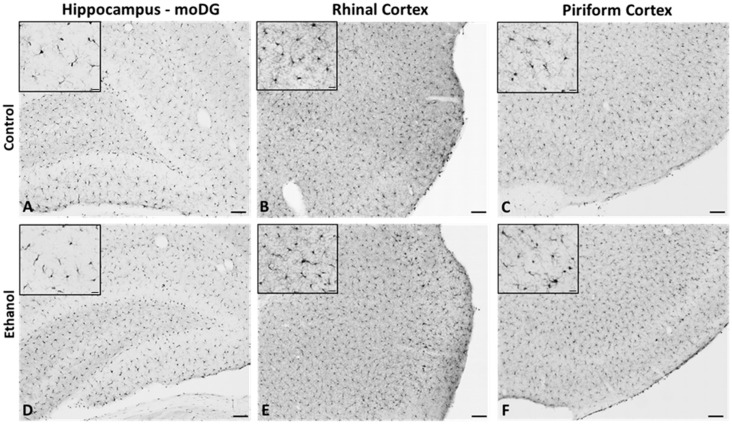
Representative images of Iba1 immunoreactivity in hippocampus (**A**,**D**), rhinal cortex (**B**,**E**), and piriform cortex (**C**,**F**) of control (top-panel) and ethanol (bottom-panel) diet-treated rats. Scale bar: larger image 100 μm and insert 20 µm.

**Figure 2:**
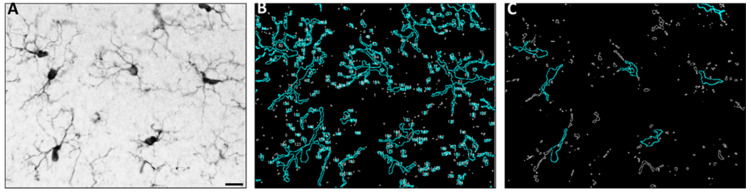
Representative images from the molecular layer of the dentate gyrus in the hippocampus showing pre-processing and thresholding strategy. Representative image of IBA1 immunoreactivity prior to thresholding; scale bar 20 µm (**A**). The two threshold levels set for images display total cells (**B**) and somas only (**C**). The analyze particles function was used to quantify the area for each image, as represented in (**B**) and (**C**) by a blue overlay.

In order to assess microglia reactivity in females after alcohol dependence, the morphology, density, and number of Iba1-immunoreactive (Iba1+) microglia were examined. Microglial reactivity is characterized by shifts in morphology that range from increased process ramification to the retraction of processes and an increase in soma size. Iba1 immunoreactivity in the hippocampus (the molecular layer of the dentate gyrus), rhinal cortex, and piriform cortex (Figure 1) was analyzed with ImageJ to assess changes in microglial morphology and number at 2 and 7 days following the final dose of ethanol (Figure 2).

**Figure 3:**
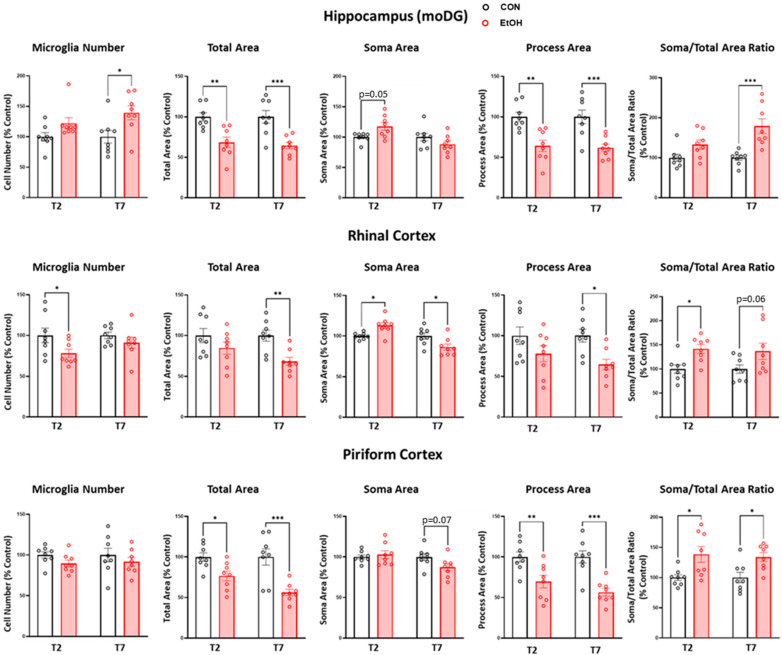
Microglia estimated number and morphology analyses in the hippocampus (moDG: molecular layer of the dentate gyrus), rhinal cortex, and piriform cortex two and seven days following control (black) and ethanol (red)-treated rats. A two-way ANOVA with post-hoc Bonferroni-corrected treatment comparisons was performed *** *p* < 0.001, ** *p* < 0.01, * *p* < 0.05.

Comparisons between the control and ethanol-treated rats at each time point demonstrated region- and dose-specific effects on microglia metrics (Figure 3), according to the two-way ANOVA with factors of diet treatment and time point, followed by planned Bonferroni post-hoc tests. In the hippocampus, the main effect of ethanol treatment [F(1,28) = 10.21, *p* = 0.0034] was driven by an increase in the number of microglia in ethanol-treated rats relative to controls at the T7 time point (*p* = 0.015). The main effects of treatment for both the total microglia area [F(1,28) = 31.04, *p* < 0.0001] and process size [F(1,28) = 31.81, *p* < 0.0001] reflected decreases in ethanol-treated rats at T2 (*p* = 0.0019; *p* = 0.0012) and T7 (*p* = 0.0005; *p* = 0.0006) relative to controls, respectively. A main effect of the treatment was revealed for the soma-to-total microglia area ratio [F(1,28) = 21.41, *p* < 0.0001], driven by an increase in the ratio in ethanol-treated rats at the T7 time point only (*p* = 0.0002).

In the rhinal cortex, a main effect of treatment was revealed for the number of microglia [F(1,28) = 5.71, *p* = 0.024] due to a decrease in the number of microglia at T2 in ethanol-treated rats versus controls (*p* = 0.046). At T7, the main effects of treatment for the total microglia area [F(1,28) = 10.74, *p* = 0.003] and process area [F(1,28) = 10.47, *p* < 0.0031] reflect decreases in the ethanol-treated rats relative to controls (*p* = 0.009, *p* = 0.018 respectively). For the soma area, a main effect of time point and a treatment x time point interaction were observed, with the post-hoc tests indicating an increase in soma area at T2 (*p* = 0.022) but a decrease at T7 (*p* = 0.014). When we analyzed the soma size to total microglia area ratio, only a main effect of diet treatment was significant [F(1,28) = 11.82, *p* = 0.002], which reflected an increase in the ratio at T2 in the ethanol group (*p* = 0.031).

In the piriform cortex, there were no changes in the number of microglia, but a main effect of ethanol treatment was apparent for the total microglia area [F(1,28) = 25.07, *p* < 0.0001] and process size [F(1,28) = 29.87, *p* < 0.0001], which were decreased at both time points in the ethanol-treated rats (area *p* = 0.041, *p* = 0.0002; process size *p* = 0.007, *p* = 0.0002). The soma-to-total microglia area ratio also demonstrated a main effect of ethanol treatment [F(1,28) = 16.31, *p* = 0.0004], which indicates that the ratio was increased in the ethanol-treated group relative to controls at both time points (T2 *p* = 0.011; T7 *p* = 0.024).

**Figure 4:**
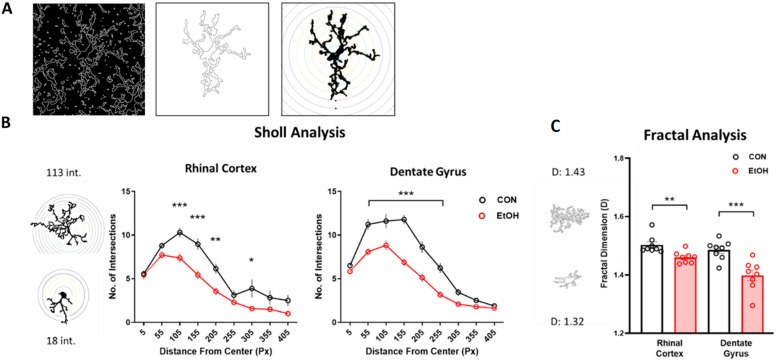
Alcohol blunts the complexity of microglia branching (morphology). Representative images (T7; 40X; Iba1+) showing microglia reconstruction (**A**). The image was thresholded and the find edges function in ImageJ was used to create an outline of all microglia. A cell was selected, and broken processes joined by hand to create both outlined and filled cells. Sholl analysis to quantify microglia branching complexity in control (black) and ethanol (red) diet-treated rats (**B**, ImageJ plugin). Representative images of greatest and least number of intersections (“int”). Results of complexity analysis in control (black) and ethanol (red) diet-treated rats (**C**; Fraclac for Fiji). Representative images of greatest and least complexity (“D”). Cells were pooled for each subject. Sholl: two-way ANOVA with Bonferroni-corrected comparisons; fractal analysis: *t*-test in each region. *** *p* < 0.001, ** *p* < 0.01, * *p* < 0.05.

The morphology analyses presented in Figure 2 and Figure 3 allow for the detection of small changes in many microglia over a large area. To extract individual microglia metrics, a single-cell morphology analysis was conducted on reconstructed microglia in a subset of the sample. Two measurements of microglial complexity, Sholl and fractal analyses, both indicated a decreased complexity in response to ethanol exposure (Figure 4). Specifically, the results of the Sholl analysis, which assesses branching complexity, highlighted decreased numbers of intersections in the dentate gyrus [F(1,126) = 111.57, *p* < 0.0001] and rhinal cortex [F(1,109) = 42.66, *p* < 0.0001] in ethanol-treated rats relative to controls. The fractal dimension, a cellular complexity measure, was similarly decreased in ethanol-treated rats in the dentate gyrus (*p* = 0.001) and rhinal cortex (*p* = 0.005).

### 2.3. Microglial Functional Phenotype Markers after a 4-Day Binge Ethanol Exposure in Adult Female Rats

**Figure 5:**
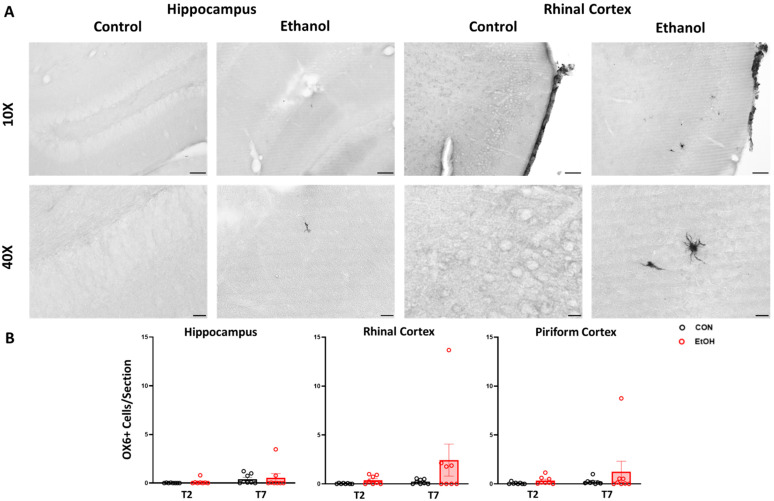
Representative OX-6/MHCII images in the rhinal cortex of control and ethanol diet-treated rats at T2 (**A**). OX-6 cell count data for the hippocampus, rhinal cortex, and piriform cortex in control (black) and ethanol (red) treated-rats (**B**). No significant differences were found via two-way ANOVA. Scale bars: top row 100 µm, bottom row 20 µm.

To determine the functional phenotype of reactive microglia, immunohistochemistry was carried out for the proinflammatory phenotype-associated cell surface markers OX-6/MHCII and ED-1/CD68. Profile counts for OX-6/MHCII positive cells showed staining throughout a number of brain regions in the ethanol-treated subjects, with high between-subject variability. The greatest number of OX6+ positive cells per section were seen in the rhinal cortices, though no comparisons were statistically significant when examined via the two-way ANOVA (Figure 5). We note here that, given the high variability in expression, this analysis is likely underpowered to draw a conclusion regarding the effects of ethanol exposure on OX-6/MHCII. In addition to the regions of interest examined here, there was significant staining noted in the cingulate, motor, sensory and insular cortices, and the amygdala.

**Figure 6:**
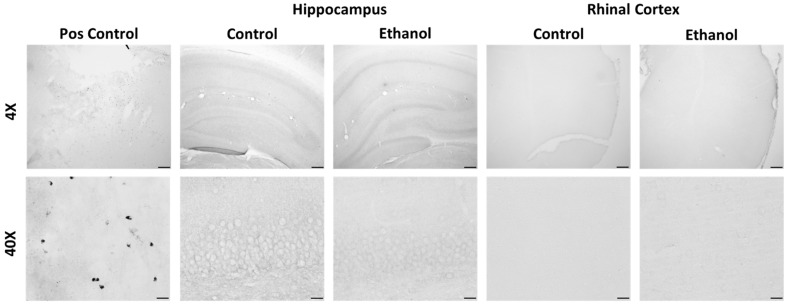
Representative ED-1/CD68 images at 4X and 40X. Positive control staining is from a stroke model rat in the primary motor cortex. In our control and ethanol-treated rats there was no positive ED-1 staining throughout the brain. Shown here are the primary regions of interest: the hippocampus and the entorhinal cortex, at the T7 time point. Scale bars: top row 200 µm, bottom row 20 µm.

Unlike OX-6/MHCII, there was no immunopositive ED-1/CD68 staining in either the control or ethanol-treated rats (Figure 6). Thus, the histology for these two functional markers indicates that the vast majority of microglia prior to or following exposure to ethanol do not exhibit a proinflammatory phenotype characterized by expression of the antigen presentation molecule MHCII and/or the phagocytosis protein CD68.

### 2.4. Changes to Neuroimmune Markers after a 4-Day Binge Ethanol Exposure

**Figure 7:**
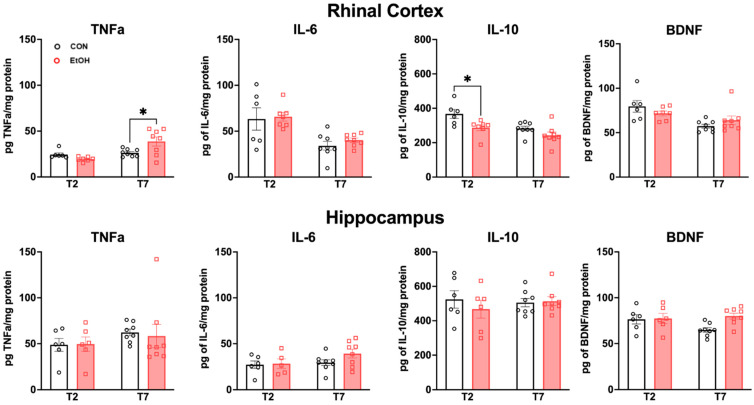
ELISA assays for proinflammatory neuroimmune markers TNFα and IL-6, and anti-inflammatory markers IL-10 and BDNF in control (black) and ethanol (red)-treated rats. In the rhinal cortex there was more TNFα in the ethanol group compared to controls at T7 (*p* = 0.0105). There was also significantly less IL-10 detected in the ethanol group compared to the controls at T2 (*p* = 0.0176). Two-way ANOVA with Bonferroni post-hoc comparisons. * *p* < 0.05.

In order to further assess the neuroimmune response to alcohol dependence, tissue cytokine and growth factor levels were examined via ELISA. The secretion of proinflammatory cytokines (such as TNFα or IL-6) occurs in microglia that exhibit a proinflammatory phenotype [37,57]. However, microglial activation can also facilitate protective functions that limit damage and aid in repair. Microglia that are in this type of activation state produce higher levels of anti-inflammatory cytokines and growth factors (such as IL-10 or BDNF) [37,57]. A two-way ANOVA did not reveal any significant differences among groups in the hippocampus (Figure 7). However, in the rhinal cortex, there was a significant ethanol treatment x time (T2 vs. T7) interaction for TNFα [F(1,25) = 8.269, *p* = 0.0081], along with a main effect of time [F(1,25) = 12.03, *p* = 0.0019]. Bonferroni post-hoc tests revealed a significant increase in the ethanol group versus control at T7 (*p* = 0.0105). There was a main effect of ethanol treatment for IL-10 [F(1,25) = 1.11, *p* = 0.0039], with Bonferroni post-hoc *t*-tests showing a significant decrease in IL-10 for the ethanol group at T2 (*p* = 0.0176). Additionally, there was an overall main effect of time in the rhinal cortex for TNFα [F(1,25) = 12.03, *p* = 0.0019], IL-6 [F(1,25) = 19.26, *p* = 0.0002], IL-10 [F(1,25) = 11.48, *p* = 0.0023], and BDNF F(1,25) = 12.35, *p* = 0.0017]. Taken together, these results indicate time- and region-specific changes in the rhinal cortex, including the early suppression of IL-10 and a latent increase of TNFα following binge ethanol exposure.

## 3. Discussion

Little is known about the consequences of excessive alcohol drinking or alcohol dependence on the female brain, especially the nature of ethanol-induced microglial reactivity. Here, we have carried out a histological analysis of microglial form and expression of functional markers, as well as an assessment of bulk tissue cytokine/growth factor protein levels in female rats that underwent a 4-day binge model of alcohol dependence. We demonstrate that in female rats treated with ethanol, microglia are reactive, yet do not demonstrate a proinflammatory phenotype based on either morphology or surface marker expression. This lack of an inflammatory signature is consistent with previous reports in male rats using the same model of alcohol dependence [42,43], as well as proteomic analyses in adult mice [58] and rat microglia [59]. Interestingly, more MHCII positive cells were noted in females than previously seen in males, which may suggest an increased sensitivity to ethanol-induced neuroimmune reactivity. These histological measures generally aligned with what we observed for key cytokine and growth factors in tissue homogenates, but also suggest a slightly greater neuroimmune response in females. For example, in the rhinal cortices, a region that is highly vulnerable to ethanol-induced damage in this model in males [29,42], as well as females [60], the proinflammatory cytokine TNFα was significantly increased at T7, while the anti-inflammatory cytokine IL-10 was decreased at T2. These two effects suggest that during this time of peak ethanol-induced toxicity and glial reactions, a more proinflammatory environment may be present in females.

For microglia, there is a well-documented relationship between morphology and function [55,61]; therefore, the first set of analyses assessed alterations in the number and morphology of microglia with ethanol treatment. Two days following the cessation of ethanol exposure, the number of microglia was decreased in the rhinal cortex relative to the controls, whereas after one week, there was an increase in the number of microglia in the hippocampus. This pattern of response appears to be slightly protracted relative to that in male rats, where microglia loss is seen in the hippocampus and rhinal cortex immediately following the cessation of ethanol after 4 days of binge-like exposure in adult males [62]. This loss persists in the prefrontal cortex of males but is not observed in females [35]. The loss of microglia is consistent with that reported for adolescent male mice as well [63]. In the hippocampus, the data complement past work, where increased numbers of microglia were noted after two and seven days of a 4-day binge exposure in male rats, as well as after 18 days of exposure in female rats [35,42,64].

With regard to morphology, the results of both the multi- and single-cell analyses highlight microglial reactivity and decreased morphological complexity according to both Sholl and fractal analyses in response to ethanol in female rats. These data complement reports of alcohol-induced changes in microglial morphology using similar methods in female adolescent rats [32], as well as in male C57Bl/6J mice (though see the discussion below; [65]). Most studies, however, have utilized a densitometry approach to measure upregulation of CD11b or Iba1 as an indicator of microglia reactivity, e.g., [42,52,66,67,68,69,70,71]. Typical densitometry methods or counts fail to tease out much of the complexity necessary to predict the cell phenotype. Shifts in morphology are subtle and challenging to quantify, e.g., [35,72]. Plus, several groups have observed reduced density of microglia [35,62,63], which may have confounded interpretation in other reports. Single-cell reconstruction can be utilized with a variety of analyses, as we have shown here, to more comprehensively assess changes to microglial morphology following ethanol exposure. The caveats are that this type of analysis is time-consuming and prone to sampling bias due to the smaller number of microglia utilized. Here, we show that, regardless of the approach taken, similar outcomes are obtained, which supports the idea that software-driven processing of multiple cells is sensitive enough to detect these subtle morphological changes seen with alcohol models. In addition, it is visibly distinct that ethanol exposure in adult female rats does not produce microglia with an ameboid morphology characteristic of a classical proinflammatory and phagocytic phenotype [73]. Instead, reactivity is more subtle. Specifically, Iba-1 immunoreactivity shows reductions in the microglial process area, branching, and cellular complexity, indicating microglial reactivity characterized by a retraction of processes (e.g., Figure 4). These morphological findings both support what we have observed in male rats and extend that work to include more nuanced measures of microglial structural changes after ethanol exposure.

Alongside morphological changes, cellular markers, such as MHCII and CD68, are indicative of specific microglial phenotypes and functions. Similar to our past work in males, few to no CD68-immunopositive (+) cells were observed in the brain parenchyma of ethanol-exposed female rats, which supports the idea that the microglial reactivity observed here does not correspond to a characteristic proinflammatory phenotype. The lack of CD68+ cells may also be due to alcohol inhibiting phagocytosis [74]. Alternatively, immunohistochemistry is insufficient in detecting the subtle changes in the expression of CD68 under conditions of mild reactivity or beneficial phenotypes, as opposed to the increased protein expression observed in brain tissue homogenates from C57Bl/6J male mice (however, see the mouse discussion below; [65]). We did note a number of OX-6/MHCII+ cells in the ethanol-treated adult female rats, particularly within the rhinal cortices. Given the high variability in OX-6/MHCII expression in the rhinal cortices of ethanol-treated rats, this one analysis may be underpowered to detect differences. This observation complements past reports, where a similarly slight, but not significant, upregulation in OX-6/MHCII+ microglia was also observed in females after alcohol but not in males [35]. The lack of observable OX-6/MHCII+ cells in males is identical to our original report in males in this model [42]. Thus, this finding is interesting, given the literature indicating increased sensitivity to the deleterious effects of ethanol in females.

Microglia release a number of cytokines and trophic factors depending on their phenotype and functional state [37,43]. In female rats, we found alcohol-induced changes in cytokine expression in the rhinal cortex that were time-specific: an increase in the proinflammatory TNFα at 7 days after the last alcohol exposure (T7), and a reduction in the anti-inflammatory IL-10 at 2 days after exposure (T2). Interestingly, no changes in cytokine/growth factor expression were observed in the hippocampus. Overall, rat studies of neuroimmune protein expression in males provide only modest evidence for alcohol-induced neuroinflammation in adults [29,42,68,75]. While the effects observed here remain modest, these results differ from what we have observed in males previously: male rats displayed decreased proinflammatory IL-6 at T2 in the rhinal cortex and increased anti-inflammatory IL-10 and TGFβ at T7 in the hippocampus [42]. Indeed, multiple binges were required to see an increase in TNFα protein expression in males [52]. In contrast, there is evidence that the female brain has more regions, particularly cortical regions, that are vulnerable to neural cell death in the same 4-day binge model [30]. Astrocyte reactivity (as measured by vimentin immunoreactivity) in females begins earlier, lasts longer, and is more widespread than that previously found in males [29,30,76]. Thus, the microglia-based effects observed here could also occur in a different timeline and in different regions for females compared to males, as observed for astrocyte reactivity [30]. In summary, these slight increases in proinflammatory cytokines, when considered with other observations of a neuroimmune response, suggest that females may show a greater proinflammatory response in the cortex compared to males [34,77]. As we focused on specific regions here, these data, when considered with other literature, may suggest a more generalized neuroimmune effect of ethanol in females [30,35,76,77] (though see [32,58]).

Our results show a change in microglial morphology coupled with modest increases in proinflammatory cytokines, contributing to a small body of literature suggesting that females may have higher baseline neuroimmune states and/or a greater inflammatory response than males [78,79]. For example, the neuroimmune mediators and cytokines, including iNOS, COX-2, and Il-1ß, were higher in female mice after alcohol exposure, which, the authors claim, underlies females’ greater susceptibility to alcohol neurotoxicity [34,80]. However, recent work suggests that adolescent male rats may be more sensitive to alcohol-induced neuroimmune and myelin effects [32]. Indeed, there are a number of sex differences in neuroimmune responses, as well as microglial reactions to insult [53,58,79,81,82]. Microglia respond in a sex-specific manner due in part to the role sex hormones play in mediating microglia functions [82,83]. Here, the variability in outcome measures was generally similar to that observed in our past work in males, which ruled out a major contribution of the estrous phase, whether considering differences between males or differences across time points within females [84]. These data, showing a subtle enhanced reaction, are consistent with microglia reactions across a variety of disease states, with females generally exhibiting an enhanced immune response compared to males (reviewed in [78,82], though see aging [53]). Thus, there is growing evidence that the same may be true for the insult of alcohol exposure in adults.

A limitation of many of these studies that reported upregulation in proinflammatory cytokine gene expression after alcohol is that they were conducted in C57BL/6J mice, which are poorly suited for immune system studies in the context of human disease [85]. Specifically, C57BL/6J mice have a mutation in the *nnt* gene, which is suspected to underlie heightened baseline neuroinflammation and oxidative stress compared to rats, as well as other mouse strains [86,87]. Indeed, this mutation renders them more sensitive to alcohol damage during development [88]. Further, this mutation in the C57Bl/6J mice likely explains the striking rat–mouse differences in alcohol effects on the neuroimmune system [89], as the C57Bl/6J are commonly used in the study of alcohol-related effects.

Another caveat in studies of microglia “activation” is that many of these studies only show that microglia react without delineating the phenotype that microglia take on. Examinations of microglia specifically, through proteomic approaches with pathway analyses or flow cytometry, do not show alcohol-induced neuroinflammatory signatures in either males or females [43,58,59,90]. Alternatively, in studies where broad panels of neuroimmune mediators have been assessed, it is clear that rats do not show classic inflammatory effects from alcohol and appear to have microglia that are pro-repair, support resolution of damage, or even exhibit a blunted response [43,90,91,92,93,94] (for a review, see [37]). Another critical function of microglia is synaptic pruning [95], which may be impacted by binge alcohol exposure [96,97]. A consequence of glia reacting to damage includes diverting microglia from their ability to perform homeostatic functions. Though we did not see the expression of the phagocytic marker CD68 at the time points examined, it is possible that this microglial synaptic remodeling is impacted by alcohol exposure, contributing to the deleterious effects of alcohol on the brain.

Genetic and preclinical studies support a relationship between neuroimmune signaling, excess alcohol consumption, and the development of an AUD [98], as reviewed in [14,39,51,99,100]. Promoting neuroinflammation is associated with increased alcohol drinking, with microglia specifically implicated in the escalation of alcohol drinking, the hallmark of an AUD [39,44]. The role of microglia in alcohol drinking is further supported by studies that genetically or pharmacologically inhibit microglia or microglia-related signaling, such as prophylactic minocycline, or enriched signals on microglia like toll-like receptors (TLRs), and observe a reduction in alcohol drinking, e.g., [98,101,102]. Most of these studies were conducted in males. Intriguingly, the one study that used females, it was observed that the LPS-induced increase in alcohol drinking persisted only in males, despite higher doses being required for the initial effect in females [103]. Poly(I:C) administration also has a differential effect in males and females due to the different timing of peak immune activation between the sexes [104,105]. Both transcriptomic (e.g., [99]) and proteomic approaches (e.g., [59]) highlight major sex differences in microglia-related signaling, as well as gaps in our knowledge of the impact of these differences on the development of AUD.

## 4. Materials and Methods

### 4.1. Binge Alcohol Model

**Figure 8:**
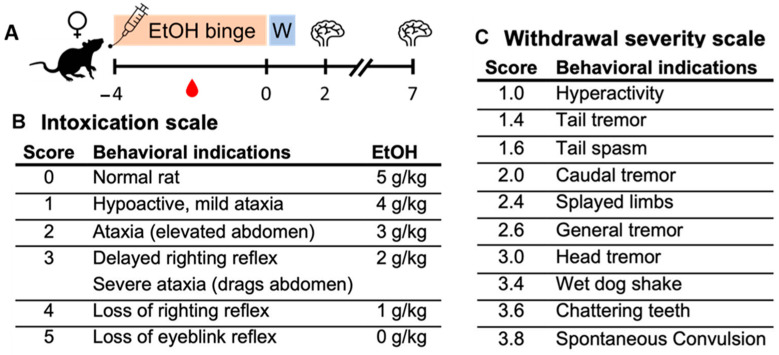
(**A**) Graphical depiction of the experimental timeline where rats were exposed to binge-like alcohol exposure for four days, then withdrew (W) from alcohol. The blood drop indicates the timing of BEC determination, and the brains indicate tissue harvest time points. (**B**) Intoxication behavioral scale and corresponding ethanol dose. (**C**) Withdrawal severity behavioral scale.

Adult, female Sprague–Dawley rats (n = 73; ~235 g; Charles River Laboratories, Raleigh, NC, USA) were pair-housed in the AAALAC-accredited vivarium at either the University of Kentucky (T7 IHC rats only) or The University of Texas at Austin (all the other rats). Rats were acclimated for five days with three minutes of daily handling by the experimenters on the last three days. Only female rats were used, as identical studies have been previously conducted and reported in males. Overlapping personnel were involved in producing the animal model at both institutions. The Majchrowicz model of alcohol dependence [106] was chosen as it has a well-described time course of neurodegenerative effects [27,28]. Critically, this model mimics the route and pattern of human consumption and high BECs characteristic of AUD [107]. Rats underwent the modified Majchrowicz model, as previously reported [30,108]. See Figure 8 for design and behavior scales and Table 1 for model parameters. Briefly, chow was removed during the binge, and every 8 h for 4 days, rats were gavaged intragastrically with an ethanol diet (25% *w*/*v* in Vanilla Ensure Plus, Abbott Laboratories, Abbott Park, IL, USA) or a control diet made isocaloric by using dextrose.

Following the initial 5 g/kg ethanol dose, subsequent doses were titrated according to a 6-point intoxication behavior scale (Figure 8B; described in [108]): rats are laid on their back and behavior is scored according to Figure 8B. Whether or how quickly the rat rights itself, and then the extent of its motoric impairments, are scored, and doses are assigned accordingly. Essentially, the less intoxicated the rat behaves, the greater the dose given. This exposure results in a high, sustained BEC > 200 mg/dL necessary for alcohol dependence, and therefore the significant neurodegenerative effects observed in this model [15]. Eight hours after the final dose, chow is returned, and all rats are moved to single housing for 24 h (“W” in Figure 8A). Beginning at 10 h following the final dose, withdrawal behaviors were observed and scored (Figure 8C) over the course of 15 h, as previously described [108]. At 24 h, rats are returned to their prior cage mate and remain until tissue collection. Blood ethanol concentrations (BECs) were determined in serum (AM1 Alcohol Analyser; Analox, London, UK) from tail blood collected 90 min following the seventh dose of ethanol. Tissue was collected 2- and 7-days following cessation of ethanol exposure, as described in the following sections. Adjacent tissue sections of some IHC rats used in this study were assessed for astrocyte markers in recent work [30]. All experiments were approved by the University of Kentucky (T7 IHC rats only) or The University of Texas at Austin (all other rats) Institutional Animal Care and Use Committee.

### 4.2. Tissue Processing and Immunohistochemistry

Rats were overdosed with sodium pentobarbital (i.p.; Fatal Plus, Vortech Pharmaceuticals, Dearborn, MI, USA) and transcardially perfused with phosphate-buffered saline, then 4% paraformaldehyde in 0.1 M phosphate buffer (pH 7.4) at 2 (T2) and 7 (T7) days following the last dose of ethanol. These time points were selected based on previous work in males, indicating that microglia demonstrate the greatest reactivity at T2, a phenomenon that persists at T7, when neuroimmune protein changes are also observed [42,43]. Additionally, the time course of cell death in females was considered [30]. Tissue harvesting and immunohistochemistry on 40 μm free-floating coronal sections followed published methods [42]. All methods, from tissue cutting forward, were conducted at The University of Texas at Austin. Immunohistochemistry was performed in batches by time point (all ethanol and all controls for a single time point run together). Every twelfth section, with sections spaced 480 μm apart, underwent diaminobenzidine-labeled (Polysciences, Waltham, MA, USA) immunohistochemistry for Iba1 (1:400; Wako, Richmond, VA, USA), a calcium-binding protein expressed in all microglia. Adjacent series of tissue were processed for two proinflammatory microglial markers: OX-6 (1:500; Biorad, Hercules, CA, USA), which binds major-histocompatibility protein class II expressed on reactive microglia (denoted as OX-6/MHCII), and ED-1, which binds CD68 (denoted as ED-1/CD68) upregulated in phagocytic microglia (1:1000; Biorad, CA, USA). For ED-1/CD68, a positive control section (ischemia model from T. Jones lab, UT Austin), was included to confirm the success of the immunohistochemistry. For all antibodies and immunohistochemistry, optimal dilutions were re-derived at The University of Texas at Austin.

### 4.3. Image Acquisition and Analysis

Iba1-stained sections were imaged using an Olympus BX43 or BX51 microscope (Olympus, Center Valley, PA, USA). Images were acquired (8 control and 8 ethanol-treated rats) in three regions across multiple bregma (3 tissue sections per subject): the dorsal hippocampus (molecular layer of the dentate gyrus; 40X; 5 images/subject), the rhinal cortex (perirhinal and entorhinal cortices combined; 20X; 3–4 images/subject), and the piriform cortex (20X; 3–4 images/subject). The images were taken using Visiopharm (Hørsholm, Denmark) and MBF Stereoinvestigator (Williston, VT, USA). These brain regions and timing were chosen based on microglia-based effects in our prior work in adult males [42,43], suggesting that these regions are primary sites of ethanol-induced damage in both males and females in this model [30]. Pre-processing and quantification of microglial morphology in Iba1-stained sections was conducted in ImageJ version 1.53, adapted from [56]. The following commands were carried out in ImageJ (batch processed for entire image set) to create thresholded images: convert 8-bit, adjust contrast, unsharp mask, set threshold, despeckle. Two threshold levels were set for images, displaying total cells and somas only. The analyze particles function was used to quantify the area for each image.

For the single-cell analyses, 40X images were acquired from the dorsal hippocampus and rhinal cortex at the T7 time point. Individual microglia (3–4 microglia per region per rat) were reconstructed prior to the Sholl and fractal analyses using ImageJ. Images were thresholded, and the ‘find edges’ function in ImageJ was used to create an outline of all microglia. A cell was selected, and broken processes joined by hand to create both outlined and filled cells. A Sholl analysis was then conducted to quantify microglia branching complexity using an ImageJ plugin. Complexity analysis was conducted using Fraclac for Fiji.

For the OX-6/MHCII- and ED-1/CD68-stained sections, profile counts were conducted across both hemispheres in all tissue sections containing the region of interest (~5–12 sections, depending on the region) in 8 control and 8 ethanol-treated rats. Counts were normalized to the number of slices containing the region of interest for each subject and are presented as counts per tissue section.

### 4.4. Enzyme-Linked Immunosorbent Assay

Rats (n = 6–8 for control and ethanol groups) were sacrificed by rapid decapitation at 2 (T2) and 7 (T7) days following the final dose of ethanol. These time points and brain regions were determined based on prior work in males for microglia reactivity [42], as well as the time course of cell death in females in this model [30]. Brains were quickly removed, and the hippocampus and rhinal cortex were dissected on ice prior to snap freezing and storage at −80 °C. For the homogenization buffer, the tissue extraction reagent (Invitrogen, Waltham, MA, USA) was combined with a protease inhibitor (Sigma, St. Louis, MO, USA) in a 10:1 ratio, respectively. This was added to the tissue (1 mL of buffer/100 mg of tissue), which was thawed and homogenized on ice using a BioSpec Tissue Tearor (Bartlesville, OK, USA). The homogenate was centrifuged at 5000 rpm at 4 °C for 10 min, and the supernatant was collected and stored at −80 °C until use. Total protein concentration was determined using a Pierce BCA Protein Assay (Thermo Fisher, Waltham, MA, USA), with samples diluted 1:8 with sterile water to fit within the standard curve of the assay. ELISA kits were used according to the manufacturer’s instructions to detect cytokine proteins: TNFα (tumor necrosis factor alpha; Invitrogen, Waltham, MA, USA), IL-10 (interleukin-10; Proteintech, Rosemont, IL, USA), BDNF (brain-derived neurotrophic factor; MilliporeSigma, Burlington, MA, USA), and IL-6 (interleukin-6; R&D Systems, Minneapolis, MN, USA). All standards, samples, and controls were run in duplicate. Cytokine concentration was divided by total protein concentration to account for differences in tissue volume and region, and results are reported as pg of cytokine per mg of protein.

### 4.5. Statistical Approaches

All statistical comparisons were carried out using Graphpad Prism version 9 (La Jolla, CA, USA). Microglial morphology metrics were averaged across the multiple sections used for each subject and calculated as percent change relative to control diet subjects. Percent control was used due to slight differences in baseline immunoreactivity among batches. For both morphology and ELISA assays, a two-way ANOVA with treatment and time as factors was carried out, and planned Bonferroni post-hoc comparisons were used to assess differences between the control and ethanol diet at each time point. Data are presented as mean ± standard error of the mean (SEM), with *p* < 0.05 accepted as significantly different.

## 5. Conclusions

In summary, the data presented here demonstrate that the neuroimmune system in female rats is subtly impacted by exposure to ethanol in a model of alcohol dependence. Moderate reactivity of microglia from ethanol-treated rats was observed in the corticolimbic regions, as demonstrated by morphological analyses suggesting an overall decreased cellular complexity due to a retraction of processes. In addition, there were no statistically significant changes in the expression of proinflammatory microglial phenotype markers, though a larger sample size is needed to confirm this lack of effect on MHCII. Cytokine and growth factor expression in corticolimbic tissue were minimally impacted by ethanol treatment. However, an increase in the proinflammatory cytokine TNFα, in addition to the presence of OX-6/MHCII+ cells in the rhinal cortex 7 days after ethanol exposure, was observed, neither of which was seen in previous work in the same model of alcohol dependence in males. The current study was designed to compare to past published work in males using the same model of exposure. Thus, one important limitation is that the study is biased towards regions and markers that were noteworthy in males, and novel results in females could have been missed. There are sex differences in the number, morphology, and reactivity of microglia to insult, including alcohol, e.g., [32,35,45,48,53,54] that might suggest that females could have a different extent and time course of microglia reactions to the insult of excessive alcohol exposure. However, our recent unbiased analysis of astrocyte reactivity and neurodegeneration shows that females are only subtly different from males in this model, and that the perirhinal and entorhinal cortices, as well as the hippocampal dentate gyrus, remain primary sites of alcohol-induced neurodegeneration [30]. Therefore, these data suggest that there may be an increased sensitivity in the neuroimmune response to ethanol exposure in female rats, underscoring the importance of including female subjects in research that seeks to understand the mechanisms of alcohol neurotoxicity and its relationship to the susceptibility and development of an AUD.

## Figures and Tables

**Table 1:** Binge subject data.

Group	*N* (C, E)	Dose (g/kg/day)	BEC (mg/dL)	Peak WD (0–4 Scale)
T2–IHC	(8, 8)	10.7 ± 0.7	402.4 ± 24.6	2.7 ± 0.4
T2–ELISA (hippocampus)	(6, 6)	11.5 ± 0.2	320.8 ± 16.8	2.1 ± 0.2
T2–ELISA (rhinal cortex)	(7, 6)	10.4 ± 0.6	403.7 ± 41.0	2.7 ± 0.5
T7–IHC	(8, 8)	10.2 ± 0.3	384.2 ± 6.9	3.3 ± 0.1
T7–ELISA	(8, 8)	10.6 ± 0.6	379.5 ± 28.4	2.2 ± 0.5

Data presented as mean ± SEM. Abbreviations as follows. C: control; E: ethanol; WD: withdrawal; T2: tissue collected 2 days after last ethanol exposure; T7: tissue collected 7 days after last ethanol exposure; IHC: immunohistochemistry; ELISA: enzyme-linked immunosorbent assay.

## Data Availability

Data will be made publicly available according to the NIH guidelines at: https://dataverse.tdl.org/dataverse/utexas (accessed on 21 November 2023).
